# EDTA as a legacy soil chelatant: a comparative study to a more environmentally sensitive alternative for metal removal by *Pistia stratiotes *L.

**DOI:** 10.1007/s11356-023-27537-6

**Published:** 2023-05-19

**Authors:** Manhattan Lebrun, Jiřina Száková, Ondřej Drábek, Václav Tejnecký, Rupert Lloyd Hough, Luke Beesley, Hailong Wang, Lukáš Trakal

**Affiliations:** 1 Department of Environmental Geosciences, Faculty of Environmental Sciences, Czech University of Life Sciences Prague, Kamýcká 129, Suchdol, 165 00 Prague 6, Czech Republic; 2 Department of Agroenvironmental Chemistry and Plant Nutrition, Faculty of Agrobiology, Food, and Natural Resources, Czech University of Life Sciences Prague, 165 00 Prague 6, Czech Republic; 3 Department of Soil Science and Soil Protection, Faculty of Agrobiology, Food and Natural Resources, Czech University of Life Sciences Prague, 165 00 Prague 6, Czech Republic; 4 The James Hutton Institute, Craigiebuckler, Aberdeen, AB15 8QH UK; 5 Biochar Engineering Technology Research Center of Guangdong Province, School of Environmental and Chemical Engineering, Foshan University, Foshan, 528000 Guangdong China; 6 Key Laboratory of Soil Contamination Bioremediation of Zhejiang Province, Zhejiang A&F University, Hangzhou, 311300 Zhejiang China

**Keywords:** Soil chelatants, DOC leaching, Metal speciation modeling, Rhizofiltration, Water lettuce

## Abstract

The accuracy of environmental risk assessment depends upon selecting appropriate matrices to extract the most risk-relevant portion of contaminant(s) from the soil. Here, we applied the chelatants EDTA and tartaric acid to extract a metal-contaminated soil. *Pistia stratiotes* was applied as an indicator plant to measure accumulation from the metal-laden bulk solutions generated, in a hydroponic experiment lasting 15 days. Speciation modeling was used to elucidate key geo-chemical mechanisms impacting matrix and metal-specific uptake revealed by experimental work. The highest concentrations of soil-borne metals were extracted from soil by EDTA (7.4% for Cd), but their uptake and translocation to the plant were restricted due to the formation of stable metal complexes predominantly with DOC. Tartaric acid solubilized metals to a lesser extent (4.6% for Cd), but a higher proportion was plant available due to its presence mainly in the form of bivalent metal cations. The water extraction showed the lowest metal extraction (e.g., 3.9% for Cd), but the metal species behaved similarly to those extracted by tartaric acid. This study demonstrates that not all extractions are equal and that metal-specific speciation will impact accurate risk assessment in soil (water)-plant systems. In the case of EDTA, a deleterious impact on DOC leaching is an obvious drawback. As such, further work should now determine soil and not only metal-specific impacts of chelatants on the extraction of environmentally relevant portions of metal(loid)s.

**Supplementary Information:**

The online version contains supplementary material available at 10.1007/s11356-023-27537-6.

## Introduction

An environmentally friendly and cost-effective solution to remediate soils or waters contaminated by metal(loid)s is to extract the pollutants using plants (often referred to as phytoextraction or rhizofiltration) (Awa and Hadibarata 2020). The quantity of metal(loid)s that a plant will take up is not only dependent on the plant biomass and capacity to uptake and translocate metal(loid)s but also on the availability of the metal(loid)s in the soil, for which soil properties, such as pH, organic matter content, and texture, are major influencing factors. To increase metal(loid) availability and thus the potential uptake by plants, chelating solutions can be applied to the soil (Ali et al. 2020; Liu et al. 2022). Much research has focused on the use of synthetic chelating agents, especially EDTA (ethylenediaminetetraacetic acid), to augment plant metal(loid) removal (Hasan et al. 2019) under the pretext that applying EDTA can increase metal(loid) mobility and render an increased bioavailability. For example, increases in the net uptake of metal(loid)s of 20 to 110% have been reported (Yuan et al. 2007; Liu et al. 2008; Shahid et al. 2011; Ali et al. 2020). However, EDTA, like most synthetic chelatants, has several disadvantages: (i) it has a high cost, (ii) is poorly biodegradable, (iii) can lead to secondary pollution (Liu et al. 2022), and (iv) can have negative impacts on plant growth (Liu et al. 2008). Alternatives addressing some or all of these concerns are low molecular weight organic acids (LMWOA) (Lu et al. 2013), which are naturally secreted by plants and microorganisms. Organic acids also have the advantage of being cheaper and biodegradable (Veselý et al. 2012a; Lu et al. 2013; Tao et al. 2020) compared to synthetic chelatants. Several studies have evaluated the impact of exogenous application of LMWOA on metal(loid) mobility and accumulation in plants, demonstrating an increase in metal(loid) mobility and uptake. For instance, when diverse organic acids (oxalic acid, citric acid, vanillic acid, and gallic acid) were applied to a metal(loid)-enriched soil, shoot, and root, Cd, Pb, Zn, Cu, and Ni concentrations in Indian mustard plants grown on that soil increased 1.4 to 10 times (do Nascimento et al. 2006). In another study by Veselý et al., (2012a), water lettuce removed 95% of Pb from a control spiked solution and 97% and 99% when citric acid and tartaric acid were added to the solution, respectively. The hyperaccumulator *Sedum alfredii* has also shown increased Cd accumulation through the exudation of tartaric acid (Tao et al. 2020). When known metal(loid)-tolerant and metal(loid)-accumulating species are tested, studies have demonstrated favorable outcomes. For example, the aquatic macrophyte *Pistia stratiotes* L. (common name: water lettuce) showed tolerance and accumulation capacity toward various metal(loid)s*. Pistia stratiotes* is a free-floating macrophyte found in most climatic regions, with a tolerance to a wide range of pH and temperature; it can form dense floating mats covering the entire water surface (Sherbeny et al. 2015; Galal et al. 2018; Ali et al. 2020) being able to grow in the presence of up to 20 ppm Cd (Das et al. 2014). This plant can also accumulate high concentrations of Cr, Cu, Fe, Mn, Ni, Pb, and Zn (Lu et al. 2011). Moreover, rhizofiltration studies evaluated its removal potential to be up to 4 g Cd.m^−2^.y^−1^, 193 g Pb.m^−2^.y^−1^, 2.2 kg Fe.m^−2^.y^−1^, 426 g Cr.m^−2^.y^−1^, 129 g Cu.m^−2^.y^−1^, 77 g Zn.m^−2^.y^−1^, and 18 g Ni.m^−2^.y^−1^ (Veselý et al. 2011; Galal et al. 2018), making water lettuce an interesting phytoextractor plant. For instance, Singh et al. (2021) showed that this plant species was able to reduce the metal load in contaminated effluents, while Şentürk et al. (2023) showed that it was efficient to remove Ni and Cr from wastewater, and Tang et al. (2020) demonstrated its capacity for Cu removal from surface water.

This study follows a previous study published in 2012 (Veselý et al. 2012b), which focused on the secondary elements present in the soil, i.e., Al, Fe, and Mn. In this paper, Cd, Cu, Pb, and Zn are the pollutants of focus because of their contrasting toxicities (Pb–Cd > Cu–Zn) and mobilities (Cd–Zn > Pb–Cu).

We hypothesized that the use of chelatants EDTA and tartaric acid will (i) increase the solubilization of metals Cd, Cu, Pb, and Zn from the soil solution compared to deionized water and (ii) modify metal speciation, which will (iii) affect the accumulation of those elements in *Pistia stratiotes*.

## Material and methods

### Soil sampling and solution extraction

The contaminated soil was sampled from an alluvial plain in the vicinity of the former heavily industrialized city of Přibram (Czech Republic) (GPS coordinates: 49°43′13 N and 14°0′46 E). This site bears a legacy of the historic atmospheric deposition of Pb via smelting activities and, latterly, the reprocessing of heavy metals from batteries. Previous studies at this site (for example, Michálková et al., 2016; Teodoro et al., 2020; Trakal et al., 2011) have resulted in a body of knowledge on the solubility of metals in soils, indicating that Pb and Zn in particular are present in pore waters at concentrations greater than would be expected, even from technogenic soils and substrates in the vicinity of mine areas (Moreno-Jiménez et al. 2011). As such this soil was chosen to be a suitable medium from which to extract metal-laden solutions in bulk. Soil sampling was carried out in a W-formation in the field, obtaining and then bulking a representative sample of topsoil (0–30 cm). After sampling, the soil was air-dried and sieved to <2 mm prior to characterization (Table 1a). To extract bulk solutions soil (1 kg) was mixed with an extraction solution (10 L) for 24 h, with an end-over shaking and under room temperature. The extraction solutions were (A) deionized water, (B) deionized water with 10 mM kg^−1^ soil tartaric acid, and (C) deionized water with 3 mM kg^−1^ soil sodium EDTA (written EDTA thereafter). After the 24-h shaking, solutions were recovered through overnight decantation and filtration. Each condition (A, B, C) was replicated three times.

**Table 1:** Chemical properties of the initial soil (a) and of the initial solutions (b) after extraction with deionized water (DW), tartaric acid (TA), and Na-EDTA (EDTA)

Soil properties (a)															
pH* (-)	CEC* (mmol kg^−1^)	TOC* (%)	DOC* (mg kg^−1^)	Available content of nutrients* (mg kg^−1^)				*N*_min_* (mg kg^−1^)	Total metal content* (g kg^−1^)						
				Ca	K	Mg	P		Al	Fe	Mn	Pb	Zn	Cd	Cu
5.7	134 ± 3	3.72	146	995 ± 68	155 ± 4	125 ± 1	58.8 ± 1	80.5	9.0 ± 0.5	20.6 ± 0.1	3.4 ± 0.3	2.5 ± 0.1	4.3 ± 0.1	0.04 ± 0.01	0.07 ± 0.01
Soil solution properties (b)															
	DOC	Ca	K	Mg	Na	NO_3_–N	NH_4_–N	Al	Fe	Mn	Pb	Zn	Cd	Cu	
DW	5.1 ± 0.3	12.6 ± 0.1	2.14 ± 0.04	0.84 ± 0.01	1.57 ± 0.02	0.18	0.27	134 ± 2	102 ± 1	2.48 ± 0.07	7.6 ± 0.1	27.8 ± 0.2	0.157 ± 0.02	0.258 ± 0.006	
TA	5.1 ± 0.1	22.4 ± 0.3	2.41 ± 0.04	1.33 ± 0.01	1.42 ± 0.02	0.01	0.03	140 ± 1	109 ± 1	2.83 ± 0.02	8.1 ± 0.1	31.0 ± 0.2	0.185 ± 0.001	0.265 ± 0.001	
EDTA	24.7 ± 0.6	15.8 ± 0.1	2.56 ± 0.07	1.32 ± 0.02	8.76 ± 0.11	0.27	0.39	102 ± 2	76 ± 1	1.82 ± 0.01	7.6 ± 0.1	32.1 ± 0.3	0.295 ± 0.002	0.251 ± 0.004	

All concentrations of selected elements in the soil leakages are in mg L^−1^

*Analytical methods described in Veselý et al. (2012b)

### Hydroponic plant growth experiment

Aquatic macrophyte (*Pistia stratiotes* L.), obtained from the Botanical Garden of Charles University (Prague, Czech Republic) (GPS coordinates: 50°04′16 N and 14°25′7 E), was selected as a suitable metal(loid)-tolerant species (Veselý et al. 2011, 2012b).

Nine pots (5 L capacity) were prepared containing 1 L of each of the three solutions detailed above (total of 27 combinations). Those nine pots corresponded to nine sampling times, i.e., 0, 1, 2, 4, 8, 24, 72, 168, and 360 h. Each pot contained 40–50 g of plant fresh weight. Over the entire experimental time course, solution pH was maintained at pH 5 using hydrochloric acid, to ensure the solubility of the metals and evaluate the effects of the modification of the speciation of the metals by the extractants on its uptake by plants.

At each sampling time, solutions and plants were collected, except for the first time (0 h) which corresponded to the initiation of the experiment (plant introduced into the pots) and for which only the solution was collected. The solutions were analyzed for total metal risk concentrations (Cd, Cu, Pb, Zn); total nutrient concentrations (Ca, K, Mg, Na), using ICP-OES (optical emission spectrometry with inductively coupled plasma) (720-ES, Varian Inc., CA, USA); total dissolved organic carbon (DOC) content using total organic carbon analyzer TOC-L a (CPH/CPN, Shimadzu), TNM-L segment flow analyzer (Shimadzu); and anion (fluoride, chloride, sulfate, phosphate, nitrate, ammonium) and organic acid (acetate, malate, formate, lactate, oxalate) concentrations using ion chromatography (ICS 6000 and ICS 90, Thermo Scientific Dionex, USA). In addition, the speciation of Cd, Cu, Pb, and Zn changes in time as well as according to the selected chelates that were determined using the Visual MINTEQ software. For this modeling, solution pH, metal and nutrient concentrations, organic acid concentration, and the forms of all detected anions were used as an input data.

All the procedures ensuring laboratory QC and QA were done according to the standard laboratory procedures including blanks and fortified standards referential materials.

Plants were harvested, and roots were cleaned with 5 mM CaCl_2_ solution to remove the metals sorbed onto the root surface (Macfie and Welbourn 2000; Veselý et al. 2011). Measurement of the metal concentration in the cleaning solution using ICP-OES allowed the determination of the concentration of metals sorbed on the root surface. After that, leaves and roots were separated and dried. The concentrations of metals in the plant tissue were determined by ICP-OES. Translocation factors (TF) and bioconcentration factors (BCF) were calculated using Eqs. (1) (Bedabati Chanu and Gupta 2016) and (2) (Rezapour et al. 2019), respectively.

1 $$\textrm{TF}=\frac{\textrm{concentration}\ \textrm{in}\ \textrm{the}\ \textrm{leaves}}{\textrm{concentration}\ \textrm{in}\ \textrm{the}\ \textrm{roots}}$$

2 $$\textrm{BCF}=\frac{\textrm{concentration}\ \textrm{in}\ \textrm{the}\ \textrm{leaves}\ \left(\textrm{or}\ \textrm{roots}\right)}{\textrm{concentration}\ \textrm{in}\ \textrm{the}\ \textrm{solution}\ \left(\textrm{initial}\right)}$$

### Statistical analysis

The concentrations of metals measured at the beginning (*t* = 0 h) and the end (*t* = 360 h) of the experiment were used to calculate extraction and removal efficiencies: (i) the extraction efficiency was calculated as the percentage of the total concentration of metal present in the soil that was removed using the extraction solution, (ii) the removal efficiency from the solution was calculated as the percentage of metal removed from the solution by the growth of water lettuce plants, and (iii) the removal efficiency from the soil mass was calculated as the percentage of the total metal concentration present in the soil that was removed by the combination of the extraction solution and plant growth.

The data were analyzed using the R software version 4.2. After testing normality (Shapiro’s test) and homoscedasticity (Bartlett’s test or Fligner’s test) of the data, means were compared using either the ANOVA test or the Kruskal-Wallis test, for parametric and non-parametric data, respectively, followed by a post hoc test. The difference was considered significant at *p* < 0.05.

## Results

### Solution composition over time

The elemental composition of the solutions after the extraction process is shown in Table 1b. The concentrations of metals measured in the solutions before plant introduction, i.e., *t* = 0 h, showed that, compared to deionized water, using 10 mM.kg^−1^ tartaric acid extracted 18% more Cd, 11% more Zn, and 7% more Pb, while using 3 mM.kg^−1^ EDTA extracted 88% more Cd and 15% more Zn. Except for Cu (for which the tartaric solution induced a higher extraction rate from the soil compared to the EDTA solution (6%)), higher concentrations of metals were measured when EDTA was used as the extractant, despite the Na-EDTA solution being less concentrated than the tartaric acid. The concentration of dissolved organic matter was higher in the EDTA solution than in the tartaric acid treatment and the deionized water. The solubilization of the nutrients (Ca, K, Mg, Na) by the different solutions was dependent on the extraction solution and the element. In more detail, calcium was measured in higher concentrations in the tartaric acid treatment and the EDTA treatment compared to the deionized water, with 77% and 25% higher concentrations than in deionized water, respectively. Magnesium was found in similar concentrations in the treatments with tartaric acid and EDTA, which had 60% higher concentrations of Mg compared to the deionized water treatment. The concentrations of potassium were highest in the EDTA treatment, followed by tartaric acid and finally deionized water. Tartaric acid did not affect the concentrations of sodium, while the solution of EDTA had almost 6 times higher concentrations of Na relative to the deionized water solution. Moreover, when comparing the amount measured in the soil used for the extraction and the amount measured in the 10 L extracting solutions after extraction, it can be seen that tartaric acid extracted the highest proportion of Ca (22.5%), Mg (10.6%), Al (15.6%), Fe (5.3%), Pb (3.2%), and Zn (7.2%), while using EDTA extracted the least Fe (3.7%) and Mn (0.5%) but the highest K (16.5%), Mg (10.6%), DOC (8.2%), Zn (7.5%), and Cd (7.6%) (Table 2). It can be noted that the use of EDTA was particularly aggressive toward the soil and removed essential fertility elements, such as K, DOC, and nitrogen, in the form of ammonium and nitrate.

**Table 2:** Efficiency of the different treatments to extract and remove metals from the soil

	Extraction efficiency (%)			Removal efficiency (%)					
	From soil mass			From solution content			From soil mass		
	DW	TA	EDTA	DW	TA	EDTA	DW	TA	EDTA
Cd	4.02	4.74	7.56	79.6	85.2	22.6	3.20	4.04	1.70
Cu	3.68	3.79	3.58	−23.9	38.5	−44.9	−0.88	1.46	−1.61
Pb	3.01	3.23	3.01	57.6	74.8	40.9	1.74	2.42	1.23
Zn	6.49	7.22	7.47	54.9	74.1	7.2	3.56	5.35	0.54

Extraction efficiency corresponds to the percentage of the metals present in the soil extracted by the solutions; removal efficiency from the solution content corresponds to the percentage of the metals present in the solution removed by plant growth; removal efficiency from soil mass corresponds to the percentage of total metal removed from the soil by the combination of the extraction solution and plant growth. Negative value indicates an absence of efficiency to remove the element

*DW* deionized water, *TA* tartaric acid, *EDTA* sodium EDTA

### Total metal concentrations

**Fig. 1:**
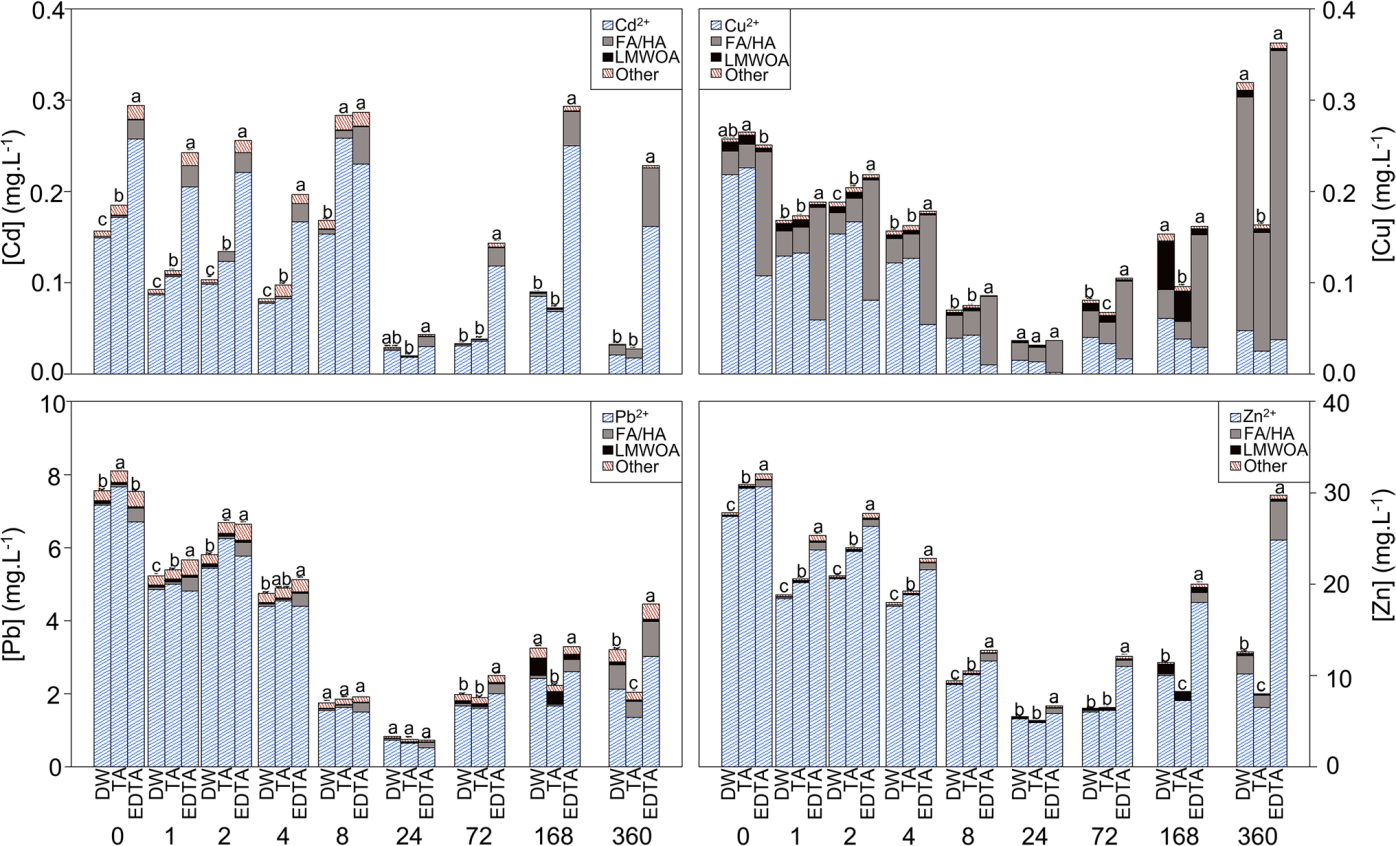
Concentrations of the different metal forms (assessed by the visual MINTEQ model) in the different growing solutions over the 360 hours of the experiment. Letters indicate significant differences in total metal concentration between treatments at each sampling time (*n* = 3) (*p* < 0.05). DW, deionized water; TA, tartaric acid; EDTA, Na-EDTA

Over the 360 h of the experimental time course, concentrations of Cd in solution varied greatly, with the concentrations decreasing in the order EDTA > tartaric acid > deionized water. For Pb, Cu, and Zn, concentrations decreased from 0 to 24 h and then slightly increased, with the highest concentrations measured in the EDTA treatment (Fig. 1).

At the end of the experiment (*t* = 360 h), Cd was higher in the EDTA solution (7-fold) than in the tartaric acid and the deionized water treatments. The concentrations of copper were similar in the EDTA solution and the deionized water but lower in the tartaric acid. Concentrations of lead and zinc decreased in the order EDTA > deionized water > tartaric acid. The comparison of the concentrations of metals between the initial (*t* = 0 h) and the final (*t*= 360 h) samplings showed that the growth of water lettuce in the deionized water removed 80%, 58%, and 55% of Cd, Pb, and Zn (Table 2), respectively. In comparison, its growth in the tartaric acid solution removed 85%, 39%, 75%, and 74% of Cd, Cu, Pb, and Zn, respectively, and only 23%, 41%, and 7% of Cd, Pb, and Zn when plants grew in the EDTA solution (Table 2). When associating this with the content of metals in the initial soil, we can see that tartaric showed the best removal efficiency, for the four elements (Table 2).

### Metal speciation and DOM concentration in the solutions

The concentrations of dissolved organic matter were higher in the EDTA solution than in the tartaric acid treatment and the deionized water throughout the experimental time course (Table S1). At the end of the experiment (*t* = 360 h), compared to the deionized water, the concentration of DOM was on average 57% higher in the EDTA and 30% lower in the tartaric acid treatment.

Using the chemical composition of the growing solutions, the Visual MINTEQ software was used to model the forms of the metals found in the solution. These forms were grouped into four categories: (i) free ions (Cd^2+^, Cu^2+^, Pb^2+^, Zn^2+^), (ii) the metals linked to fulvic and humic acids (FA/HA), (iii) the metals linked to LMWOA, and (iv) the metals in “other” forms (Fig. 1).

Throughout the experimental time course, Cd was mainly found as a free Cd^2+^ ion. The increase in Cd concentration in the solution with tartaric acid and especially EDTA was mainly attributed to an increase in free Cd ion and, to a lesser extent, the “other” fraction (composed mainly of CdCl^+^, CdSO_4_, CdNO_3_, and CdF^+^). In addition, EDTA greatly increased the concentration of Cd associated mainly with acids. Over the 360 h of the experimental time course, concentrations of free Cd^2+^ ions tended to decrease especially in the deionized water and tartaric acid treatments, while the concentration of Cd associated with fulvic and humic acids increased with time in the EDTA treatment. This implies that Cd consumption by plants was mostly through the uptake of free Cd^2+^ ions.

At the initial time (*t*=0 h), Cu was mostly present as free Cu^2+^ ions, followed by Cu associated with fulvic and humic acids and Cu associated with LMWOA and the “other” Cu forms (composed mainly of CuSO_4_, CuOH^+^, CuNO_3_^+^, CuCl^+^, and CuF^+^), in the deionized water treatment. The use of tartaric acid did not affect the relative concentrations of Cu present as different species, while EDTA decreased the concentrations of free Cu^2+^ ions and increased the fraction associated with fulvic and humic acids. Over the experimental time course, the decrease in the total concentrations of Cu was primarily due to a decrease in the concentrations of free Cu^2+^ ions, reflecting an uptake of Cu by plants via free Cu ion assimilation.

Although the concentration decreased over time, Pb was mostly present as free Pb^2+^ ions, in all three treatments. Such a decrease in the concentrations of free Pb^2+^ ions is resultant of the uptake of the Pb^2+^ ions by the plants. The second most encountered form, “other” (i.e., PbSO_4_, PbCl^+^, PbOH^+^, PbNO_3_^+^, PbF^+^), also decreased with time, while the concentrations of Pb associated with fulvic and humic acids tended to increase. The main effect of the EDTA treatment was the increase in the concentration of Pb associated with fulvic and humic acids.

Similar to the other elements, the main form of Zn in the solution was free Zn^2+^ ion, concentrations of which decreased over time in all three treatments, relative to plant uptake. The main effect of the treatment was a small increase in the concentration of Zn associated with humic and fulvic acids with EDTA, which was more important with time.

### Nutrient contents

Concentrations of nutrients tended to stay stable until hours *t* = 8 or 24, after which they decreased in all treatments. At the end of the experiment (*t* = 360 h), nutrient concentrations decreased in the order of EDTA > tartaric acid > deionized water. Compared to the concentrations measured in the solutions at the beginning of the experiment, after 360 h, plants consumed 86%, 74%, 97%, and 96% of Ca, Mg, K, and Na, respectively, in the control; 83%, 73%, 94%, and 83% in the tartaric acid; and 70%, 55%, 97%, and 80% in the EDTA treatment.

Regarding their ionic forms, the anion concentrations showed more variability through time (Table S1). At the end of the experiment, all anion concentrations decreased compared to *t* = 0, except for fluoride and sulfate. All treatments presented similar concentrations except for chloride (tartaric acid > EDTA > deionized water) and sulfate (deionized water > EDTA > tartaric acid).

Similar to the reported concentrations of anions, an important variability with time was seen in the case of organic acid (Table S1). On average, concentrations of lactate decreased in the order tartaric acid > EDTA > deionized water; acetate decreased in the order EDTA > deionized water > tartaric acid; formate in the order tartaric acid > deionized water > EDTA; and malate and oxalate decreased in the order deionized water > tartaric acid > EDTA.

This shows that there were higher concentrations of nutrients in the EDTA and tartaric solutions than in the deionized water, but the EDTA treatment extracted the most elevated concentrations of Na which could induce salt stress in plants.

### Metal accumulation in plants

**Fig. 2:**
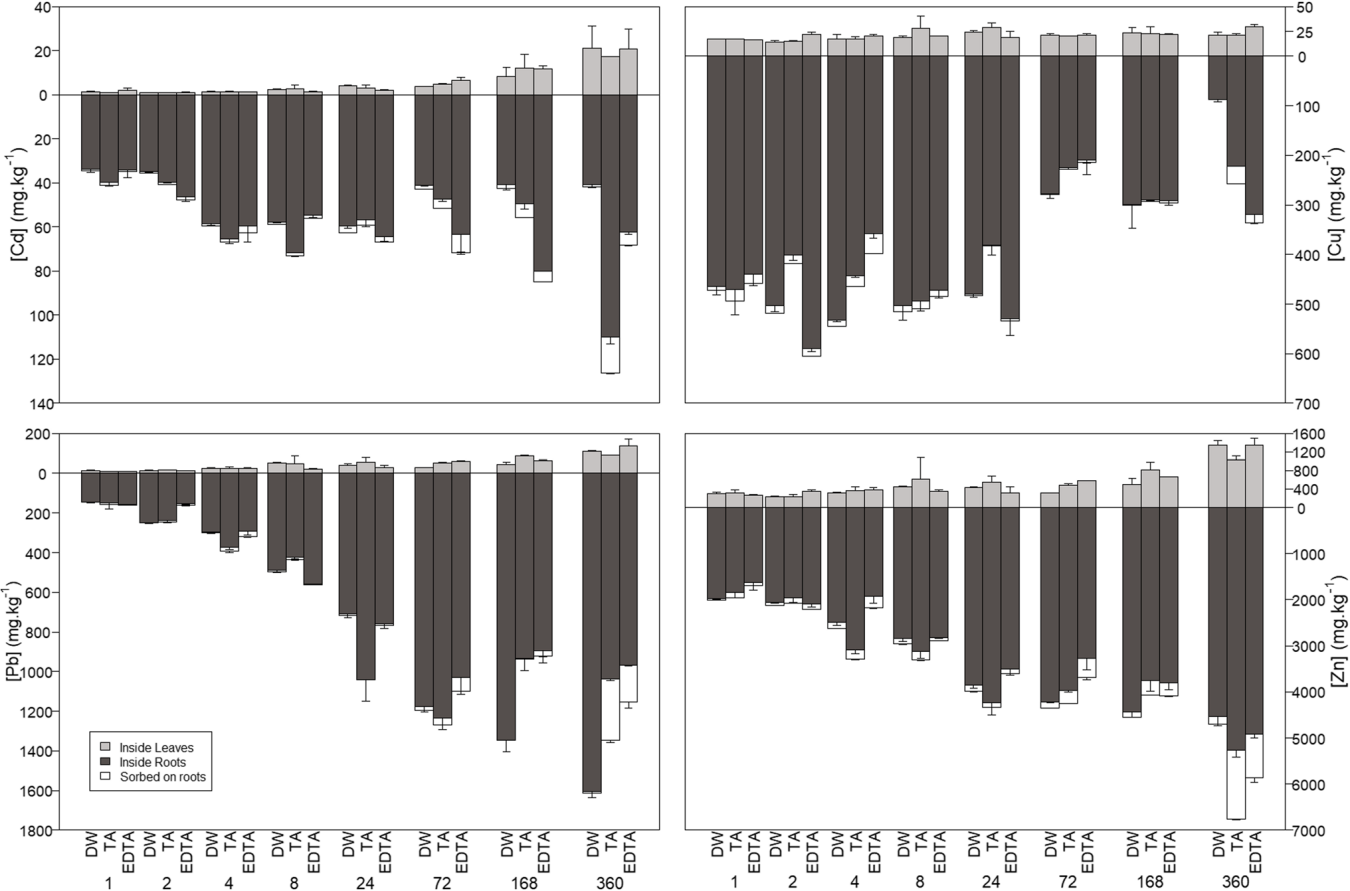
Metal concentration sorbed onto the roots and accumulated inside the leaves and inside the roots under the three treatments and over the 360 hours of the experiment. Data are presented as mean ± SD (*n* = 2–3). DW, deionized water; TA, tartaric acid; EDTA, Na-EDTA

The accumulation of Cd, Cu, Pb, and Zn onto the roots and inside the leaves and the roots of *Pistia stratiotes* was monitored over the 360 h of the experiment (Fig. 2). Most of the metals accumulated inside the roots, followed by an accumulation inside the leaves and onto the root surfaces.

The sorption of Cd onto root surfaces increased with time but generally decreased in the order tartaric acid > EDTA > deionized water (Fig. 2). The highest levels of Cd adsorption to root surfaces were measured at 24 h for the control, 360 h for the solutions containing tartaric acid, and at 72 h for the EDTA. Copper sorption onto the roots showed greater variability over both time and treatments compared to Cd, although Cu sorption was generally higher with tartaric acid solution and EDTA solution compared to deionized water and decreased with time, except at the final sampling. The highest concentrations were found at 2 h for the control, 360 h for the tartaric acid, and 4 h for the EDTA. Sorption of Pb onto the roots was low in all treatments at the beginning of the experiment and increased with time, with higher levels of sorption associated with the EDTA and tartaric acid treatments compared to the deionized water treatment. The highest concentrations were measured at 360 h for the tartaric acid and EDTA treatments and 72 h for the control. Finally, Zn sorption onto the roots increased with time and was higher when tartaric acid or EDTA was used to obtain the growing solutions than the deionized water solution. The concentration in the control did not evolve much, while the highest concentrations in the tartaric acid and EDTA treatments were measured at 360 h.

The different treatments used to obtain the growing solutions had little effect on the concentrations of Cd, Cu, Pb, and Zn in the shoot tissues, while the concentrations in the root tissues were more affected (Fig. 2). Concentrations of Cd in plant tissues were elevated in the tartaric acid and EDTA treatments relative to the deionized water treatment, while concentrations of Pb decreased with both treatments; Cu initially decreased and then increased in the treatments with tartaric acid and EDTA, and Zn was little affected by time or treatment. Except for Cu, for which concentrations in the roots decreased while concentrations in the shoots remained the same with time, the concentrations of Cd, Pb, and Zn increased with time in both shoots and roots and for all three treatments. Maximum concentrations of Cd, Pb, and Zn in shoot tissues were measured at 360 h in all treatments, while maximum concentrations of Cu in shoots were measured at 360 h only in the case of EDTA and at 24 h for the other two treatments. Maximum concentrations of Cd in root tissues were observed at 24 h for the control, 360 h for the tartaric acid, and 168 h for the EDTA treatment. Maximum concentrations of Cu in both plant tissues were measured in the initial phases of the experiment: 4 h for the control, 8 h for the tartaric acid, and 2 h for the EDTA. In the control, the maximum concentrations of Pb in root tissues occurred at 360 h, while in the other two treatments, it was earlier (72 h). Finally, in all cases, maximum concentrations of Zn in roots were measured at 360 h.

When looking at the repartition of the metals within the plants, it can be seen that the treatments “tartaric acid” and “EDTA” had mainly an effect in the latter phases of the experiment (i.e,. after 24 h) (Fig. 2). More precisely, at the last sampling, the proportion of metals sorbed on the surface of the roots increased, while the proportion in the leaves decreased, with a more pronounced effect observed in the case of tartaric acid.

The translocation factors were low in all treatments and for all elements at the beginning of the experiment (Table 3), except for Zn. They all increased over the time of the experiment but remained below 1. The treatment effect was mostly visible at the last sampling points, in which a higher TF was observed in the deionized water treatment relative to the other two treatments. The exception to this was Pb which showed the reverse. The translocation efficiency followed the mobility of the elements, rather than the essentiality, with higher TF values for Zn and Cd than Pb and Cu, regardless of the treatments. In accordance with TF, bioconcentration factors were higher in the roots than in the leaves (Table 3). Moreover, BCFs increased with time for all elements and both organs, except for Cu. On average, for both roots and leaves, the highest values of BCF were associated with the uptake of Cu, followed by Cd, Zn, and Pb which were the lowest.

**Table 3:** Translocation (a) and bioconcentration factors for leaves (b) and roots (c) in the different treatments and during the 360-h experiment

		1	2	4	8	24	72	168	360
Translocation factor (a)									
Cadmium	DW	0.039 ± 0.008	0.023 ± 0.005	0.022 ± 0.004	0.040 ± 0.004	0.067 ± 0.005	0.088 ± 0.004	0.204 ± 0.113	0.514 ± 0.230
	TA	0.021 ± 0.003	0.025 ± 0.001	0.018 ± 0.004	0.037 ± 0.023	0.051 ± 0.027	0.102 ± 0.006	0.244 ± 0.142	0.158 ± 0.004
	EDTA	0.057 ± 0.043	0.024 ± 0.003	0.024 ± 0.004	0.024 ± 0.006	0.029 ± 0.007	0.104 ± 0.034	0.147 ± 0.019	0.333 ± 0.140
Lead	DW	0.099 ± 0.010	0.060 ± 0.007	0.086 ± 0.009	0.104 ± 0.005	0.059 ± 0.012	0.023 ± 0.001	0.031 ± 0.008	0.069 ± 0.004
	TA	0.064 ± 0.012	0.070 ± 0.011	0.071 ± 0.014	0.117 ± 0.088	0.054 ± 0.016	0.042 ± 0.002	0.093 ± 0.011	0.088 ± 0.000
	EDTA	0.062 ± 0.009	0.077 ± 0.008	0.089 ± 0.010	0.038 ± 0.008	0.038 ± 0.015	0.057 ± 0.002	0.071 ± 0.002	0.140 ± 0.039
Copper	DW	0.037 ± 0.001	0.028 ± 0.005	0.033 ± 0.008	0.037 ± 0.001	0.050 ± 0.003	0.078 ± 0.002	0.080 ± 0.033	0.242 ± 0.050
	TA	0.037 ± 0.004	0.037 ± 0.002	0.039 ± 0.004	0.058 ± 0.027	0.075 ± 0.008	0.090 ± 0.001	0.078 ± 0.024	0.096 ± 0.006
	EDTA	0.037 ± 0.001	0.037 ± 0.004	0.058 ± 0.006	0.043 ± 0.001	0.035 ± 0.009	0.102 ± 0.008	0.075 ± 0.005	0.092 ± 0.003
Zinc	DW	0.155 ± 0.015	0.110 ± 0.010	0.125 ± 0.006	0.156 ± 0.005	0.114 ± 0.005	0.073 ± 0.002	0.112 ± 0.034	0.297 ± 0.009
	TA	0.171 ± 0.023	0.122 ± 0.015	0.117 ± 0.023	0.202 ± 0.158	0.129 ± 0.023	0.120 ± 0.011	0.220 ± 0.056	0.196 ± 0.011
	EDTA	0.164 ± 0.025	0.165 ± 0.008	0.201 ± 0.005	0.123 ± 0.011	0.090 ± 0.035	0.178 ± 0.011	0.173 ± 0.003	0.273 ± 0.026
Bioaccumulation factor: leaves (b)									
Cadmium	DW	8.43 ± 1.97	5.10 ± 1.07	8.28 ± 1.75	14.58 ± 1.68	25.38 ± 2.44	23.18 ± 1.25	52.20 ± 26.50	134.26 ± 64.17
	TA	4.46 ± 0.79	5.37 ± 0.24	6.32 ± 1.81	14.41 ± 8.93	15.80 ± 9.02	26.07 ± 0.99	64.53 ± 34.88	94.01 ± 0.15
	EDTA	6.32 ± 4.31	3.77 ± 0.31	4.73 ± 0.26	4.47 ± 1.18	6.28 ± 1.75	21.87 ± 4.40	39.77 ± 5.03	70.58 ± 31.03
Lead	DW	1.89 ± 0.27	1.97 ± 0.25	3.36 ± 0.33	6.72 ± 0.47	5.52 ± 0.95	3.58 ± 0.24	5.62 ± 1.66	14.72 ± 0.47
	TA	1.16 ± 0.02	2.04 ± 0.22	3.25 ± 0.76	6.16 ± 4.72	7.06 ± 2.79	6.35 ± 0.58	10.63 ± 0.65	11.28 ± 0.09
	EDTA	1.28 ± 0.19	1.57 ± 0.17	3.43 ± 0.18	2.82 ± 0.55	3.82 ± 1.58	7.78 ± 0.87	8.46 ± 0.39	17.94 ± 4.91
Copper	DW	65.91 ± 0.34	54.23 ± 8.04	67.98 ± 16.94	73.23 ± 6.10	92.95 ± 5.82	83.65 ± 5.18	90.31 ± 23.18	81.36 ± 12.62
	TA	65.48 ± 0.30	56.06 ± 3.94	65.11 ± 7.71	106.78 ± 46.26	108.88 ± 16.44	76.14 ± 0.29	85.25 ± 26.01	80.50 ± 4.57
	EDTA	65.54 ± 1.19	87.99 ± 7.48	82.28 ± 6.21	80.48 ± 1.27	75.03 ± 24.10	84.56 ± 4.90	87.02 ± 3.24	117.25 ± 10.21
Zinc	DW	10.98 ± 1.22	8.10 ± 0.61	11.19 ± 0.85	15.90 ± 0.88	15.77 ± 0.37	11.07 ± 0.29	17.82 ± 4.94	48.53 ± 3.61
	TA	10.24 ± 2.03	7.76 ± 1.29	11.68 ± 2.57	19.97 ± 14.93	17.75 ± 4.26	15.36 ± 1.50	26.47 ± 5.17	33.40 ± 2.76
	EDTA	8.28 ± 0.45	10.73 ± 0.94	12.06 ± 1.33	10.85 ± 1.03	9.87 ± 4.18	18.06 ± 0.29	20.56 ± 0.43	41.89 ± 4.73
Bioaccumulation factor: roots (c)									
Cadmium	DW	215.7 ± 7.5	222.0 ± 1.5	371.8 ± 4.4	367.2 ± 3.6	380.2 ± 6.2	262.2 ± 1.0	260.0 ± 14.5	259.2 ± 9.1
	TA	215.8 ± 7.8	214.7 ± 1.6	353.5 ± 12.5	387.6 ± 1.2	306.7 ± 16.9	255.7 ± 6.5	268.5 ± 13.1	595.8 ± 16.6
	EDTA	115.4 ± 12.0	157.7 ± 7.0	202.1 ± 24.8	185.1 ± 3.7	218.4 ± 6.9	215.1 ± 27.5	271.0 ± 1.0	210.9 ± 4.6
Lead	DW	19.0 ± 0.7	32.8 ± 0.4	38.9 ± 0.0	64.6 ± 1.4	93.8 ± 2.3	155.6 ± 3.6	178.0 ± 7.8	212.4 ± 3.9
	TA	18.5 ± 3.7	29.2 ± 1.6	45.8 ± 1.7	52.1 ± 1.0	128.2 ± 13.6	152.1 ± 7.2	115.1 ± 7.3	127.7 ± 0.8
	EDTA	20.6 ± 0.0	20.4 ± 0.1	38.6 ± 2.4	74.1 ± 0.6	100.7 ± 3.1	136.1 ± 9.3	118.5 ± 8.1	128.2 ± 0.3
Copper	DW	1803.6 ± 62.4	1950.0 ± 50.4	2068.2 ± 8.8	1955.8 ± 108.7	1862.6 ± 23.1	1078.4 ± 36.8	1161.1 ± 187.0	338.1 ± 17.9
	TA	1773.8 ± 195.6	1514.1 ± 38.7	1670.2 ± 14.1	1864.3 ± 72.1	1439.4 ± 73.9	848.9 ± 13.4	1091.5 ± 9.1	835.2 ± 1.5
	EDTA	1753.4 ± 94.9	2350.6 ± 27.0	1429.7 ± 33.9	1885.3 ± 63.0	2112.2 ± 137.0	836.1 ± 114.5	1165.0 ± 34.2	1273.7 ± 74.7
Zinc	DW	71.0 ± 0.8	73.9 ± 1.0	89.7 ± 2.3	102.1 ± 2.3	138.3 ± 2.4	151.7 ± 0.3	159.3 ± 4.3	163.1 ± 7.0
	TA	59.6 ± 3.8	63.4 ± 2.9	99.8 ± 2.5	100.8 ± 4.7	136.9 ± 8.7	128.3 ± 0.7	121.2 ± 7.5	169.9 ± 4.9
	EDTA	50.8 ± 5.1	65.0 ± 2.4	59.9 ± 5.0	88.0 ± 0.5	109.4 ± 4.0	102.0 ± 7.9	118.7 ± 4.5	153.4 ± 2.7

Data are presented as mean ± SD (*n* = 3)

*DW* deionized water, *TA* tartaric acid, *EDTA* Na-EDTA

## Discussion

Taken together, these results show that although EDTA solubilized higher concentrations of metals than tartaric acid and deionized water, at the end of the experiment, higher concentrations remained in this treatment, while tartaric acid solubilized higher metal concentrations than the control and metal removal by plants which was the highest in this treatment.

### Metal mobility as influenced by the extractants

In this study, the use of synthetic and natural chelatants was compared for the extraction of metals and their subsequent (bio)availability. It has already been well documented that chelatants are efficient for this purpose (Yuan et al. 2007; Liu et al. 2008; Veselý et al. 2012a), and the mechanisms have been identified to be the competition for sorption sites and the formation of soluble LMWOA- or EDTA-metal complexes (Yuan et al. 2007; Tao et al. 2020; Liu et al. 2022). In most cases tested here, apart from Cu, the synthetic chelatant EDTA was more efficient than the natural one (tartaric acid) at increasing the mobility of metals. This is consistent with previous studies (do Nascimento et al. 2006; Evangelou et al. 2006; Liu et al. 2008) and is related to the high chelation stability constant of EDTA (de Santiago-Martín et al. 2013). In addition to this, chelating solutions increased not only the solubility of metals but also modified their speciation. For example, the use of EDTA to extract metals induced a high concentration of metal associated with fulvic and humic acids, in addition to the high proportion of metal present as free divalent ions that were also observed for the other two chelatants/extractants. In previous studies, humic/fulvic acid complexes have been linked to the strong chelating capacity of EDTA (Yang et al. 2001; Zhang et al. 2010), which not only mobilize elements weakly bound to extant soil matrix constituents but can also extract soil organic matter (of which humic acids are a major component), releasing additional elements (bound to this fraction) into solution (Yang et al. 2001; Zhang et al. 2010, 2012). This is supported by the higher concentrations of DOM measured in the EDTA treatment relative to the control and tartaric acid treatments. The different forms of metals present in the growing media will influence their toxicity and potential for uptake by plants and biota (Chen et al. 2010). For instance, the study of Ondrasek et al., (2018) showed that the application of humic acids to the soil decreased the accumulation of Cu, Zn, Cd, and Mn in plants due to the formation of metal-humate complexes, whereas the application of EDTA increased the accumulation of Pb in *Vicia faba* plants due to the formation of metal-EDTA complexes (Shahid et al. 2014a). As speciation also evolved during the experimental time course in our study, it can be hypothesized that the presence of plants adjusted metal speciation through preferential uptake of different metal species, as well as the secretion of root exudates, which may have differed between treatments (Haoliang et al. 2007; Kidd et al. 2009) and through exposure time (Wei et al. 2023). This was exemplified in our previous study, where concentrations of both total and singular amino acids in the leaves of *Pistia stratiotes* were modified over time by the use of tartaric acid and EDTA (Veselý et al. 2012b). Although the measurements were made in the leaves in that study, it demonstrates that the metabolism of plants changed with both treatment and time. From this, it is reasonable to infer that root exudation was also modified in a similar way; thus, plant-mediated metal speciation differed between treatments and with time. This was also supported by the high variation of organic acid contents in the growing media with time in the different treatments (Table S1).

An elevated concentration of Na in the solutions obtained using the EDTA treatment could also have affected the growth of plants as well as the uptake of metals (Haddad and Al-Jada 2021).

### Changes in speciation affected the plant uptake

The concentrations of metals measured in the shoots and the roots of *Pistia stratiotes* in this study were greater than those found in previous studies using the same plant species under higher soil/solution metal concentrations (Lu et al. 2011; Veselý et al. 2012a; Galal et al. 2018; Kumar et al. 2019; Xie et al. 2022). This demonstrates the efficiency of plants to uptake metals in solution particularly using the chelatants (tartaric acid and EDTA). Tartaric acid enhanced the uptake of metals by the plants to the greatest extent. This can be explained by a higher root uptake, an increase in xylem transport, and an enhanced biomass production (Lu et al. 2013). The results of the metal uptake by plants, and removal capacity, also revealed that although EDTA increased metal solubility more than tartaric acid, it did not induce a higher removal of metals by plants. Such an effect can be attributed to speciation differences and selective uptake by plants. Previous studies showed that EDTA increased the uptake of metals by plant roots and their translocation toward the upper parts (Shahid et al. 2014b, a; Shamshad et al. 2018). This effect is usually attributed to the high concentration of soluble EDTA-metal complexes, as well as the capacity of EDTA to damage the root physiological barrier (do Nascimento et al. 2006). However, in our case, metal contents in the plant were minimally affected by the addition of EDTA, despite elevated concentrations of metals in the solution. This finding was in contrast to the work of Shamshad et al., (2018) and Bunluesin et al., (2007) but in accordance with the finding of Lambrechts et al., (2011); clearly, there is not a settled consensus on this effect. When looking at the speciation of the elements in the solution, it can be seen that tartaric acid increased the concentrations of metals in the solution primarily through the increase of free divalent ions. In contrast, and as described above, the EDTA treatment also increased the concentrations of metals associated with humic and fulvic acids. Such metal species may not be available for plant uptake and translocation, and thus, the uptake of metals by plants has been shown to be mainly related to the concentration in free Me^+^ forms in the growing media (Wang et al. 2008; Chen et al. 2010; Shabbir et al. 2020; Kumar et al. 2021). The application of humic acids has also been shown to decrease the accumulation of metals by plants (Bunluesin et al. 2007; Rong et al. 2020). The authors related such an observation to a decrease in the concentration of free metal ions in the growing solution and the formation of stable complex with metals. Such an explanation is supported by the evolution of the concentration of the metal forms through time; the concentrations of free metal ions decreased in all cases, but in the EDTA treatment, the content of metal linked to humic and fulvic acids did not decrease, indicating that such complexes were not taken up by the plants. Furthermore, even if taken up by the roots, such complexes are large molecules with reduced transport from the roots to the shoots (Wang et al. 2008; Chen et al. 2010). This could explain the lower translocation factor of Cd and Cu in the EDTA treatment. Finally, the higher amount of Ca and Mg in the EDTA treatment could have contributed to suppressing the removal of metals by the plants, as those elements are antagonists of cations for plant uptake (Kabata-Pendias 2004). In summary, tartaric acid mobilized a small proportion of metals that were readily available for plant uptake (free ion form), while EDTA solubilized a high quantity of metals that were less readily available for plant uptake (form complexed with fulvic and humic acids).

### Risks and benefits associated with the application of chelatants

In most situations where phytoextraction of metals from soils or waters is employed, the plant material is ultimately removed and used for non-food-chain purposes such as feedstock for anaerobic digestion. The main considerations are therefore the impacts of the chelator on longer-term soil and water quality (the ability of the soil to support plant growth once metals have been removed), as well as any potential the chelator might have to impair the growth of the selected accumulator crop. The fact that EDTA solubilized DOM reduces its useful application to soils because fertility will be reduced, and soil washing with this agent has been shown to be detrimental to soil structure and microbial activity (Jelusic and Lestan 2014). From the results of our study, it can also be seen that EDTA induced a higher stress on the plants, especially after 360 h, as plants contained higher concentrations of proline (stress marker) compared to the other treatments (Veselý et al. 2012b), even though metal accumulation was similar, showing that toxicity stress came from other elements than just metals, Na for instance. This brings into question the use of EDTA for soil remediation applications in the field, and our study indicates that the use of tartaric acid is a more appropriate option where soil quality needs to be preserved.

## Conclusions

EDTA ensured the highest metal extraction from the soil, but their uptake and translocation to the plant were restricted due to the formation of stable metal complexes predominantly with DOC. In the case of EDTA, a harmful impact on DOC leaching from the soil is an obvious drawback. On the other hand, tartaric acid (as well as water) solubilized metals to a lesser extent, but a higher proportion was plant available due to its presence mainly in the form of bivalent metal cations.

This study demonstrates that not all extractions are equal and that metal-specific speciation will impact accurate risk assessment in soil (water)-plant systems. To conclude, the usage of EDTA should be avoided due to its aggressive propensity to render metals soluble (leachable) but not bioavailable. The focus of future research in this area should be on the usage of tartaric acid as an environmentally relevant chelation in various soils that have been treated with remedial amendments, and tartaric acid should be tested under more complex conditions, i.e., pot and field experiments.

## Supplementary information

ESM 1

## Data Availability

The datasets generated and/or analyzed during the current study are available from the corresponding author on reasonable request.

## Abbreviations

EDTA: Ethylenediaminetetraacetic acid
LMWOA: Low molecular weight organic acid
DOC: Dissolved organic carbon
DOM: Dissolved organic matter
