# Correction to: EDTA as a legacy soil chelatant: a comparative study to a more environmentally sensitive alternative for metal removal by Pistia stratiotes L.

**DOI:** 10.1007/s11356-023-27966-3

**Published:** 2023-05-29

**Authors:** Manhattan Lebrun, Jiřina Száková, Ondřej Drábek, Václav Tejnecký, Rupert Lloyd Hough, Luke Beesley, Hailong Wang, Lukáš Trakal

**Affiliations:** 1 Department of Environmental Geosciences, Faculty of Environmental Sciences, Czech University of Life Sciences Prague, Kamýcká 129, Suchdol, 165 00 Prague 6, Czech Republic; 2 Department of Agroenvironmental Chemistry and Plant Nutrition, Faculty of Agrobiology, Food, and Natural Resources, Czech University of Life Sciences Prague, 165 00 Prague 6, Czech Republic; 3 Department of Soil Science and Soil Protection, Faculty of Agrobiology, Food and Natural Resources, Czech University of Life Sciences Prague, 165 00 Prague 6, Czech Republic; 4 The James Hutton Institute, Craigiebuckler, Aberdeen, AB15 8QH UK; 5 Biochar Engineering Technology Research Center of Guangdong Province, School of Environmental and Chemical Engineering, Foshan University, Foshan, 528000 Guangdong China; 6 Key Laboratory of Soil Contamination Bioremediation of Zhejiang Province, Zhejiang A&F University, Hangzhou, 311300 Zhejiang China

**Correction to: Environmental Science and Pollution Research**

**https://doi.org/10.1007/s11356-023-27537-6**

In the article title, it should be EDTA instead of ETDA.

The Original article has been corrected.

