# Supplementary material for: EDTA as a legacy soil chelatant: a comparative study to a more environmentally sensitive alternative for metal removal by Pistia stratiotes L
Source: Environ Sci Pollut Res Int. 2023 May 19;30(29):74314–26. doi: 10.1007/s11356-023-27537-6 (PMC10287577; doi:10.1007/s11356-023-27537-6)
Supplement: Supplementary file 1 — ESM 1 [file 11356_2023_27537_MOESM1_ESM.docx]

**Table S1.** Composition of the different growing solutions (mg.L^-1^) with time: Letters indicate significant differences between treatments at each sampling time, for the nutrients (p < 0.05) (n = 3). DW = deionized water, TA = tartaric acid, EDTA = Na-EDTA.

| **Element** | **Treatment** | **0** | **1** | **2** | **4** | **8** | **24** | **72** | **168** | **360** |
| --- | --- | --- | --- | --- | --- | --- | --- | --- | --- | --- |
| *DOC* | *DW* | 5.1 ± 0.4 b | 4.8 ± 0.2 b | 4.2 ± 0.2 b | 4.5 ± 0.1 b | 4.5 ± 0.1 b | 4.3 ± 0.1 b | 5.1 ± 0.2 b | 5.6 ± 0.2 b | 35.7 ± 0.6 b |
|  | *TA* | 5.1 ± 0.1 b | 5.1 ± 0.1 b | 4.7 ± 0.2 b | 4.9 ± 0.1 b | 4.8 ± 0.1 b | 4.5 ± 0.1 b | 5.1 ± 0.1 b | 5.8 ± 0.0 b | 24.9 ± 0.4 c |
|  | *EDTA* | 24.7 ± 0.6 a | 24.4 ± 0.4 a | 23.6 ± 0.5 a | 24.1 ± 0.6 a | 24.3 ± 0.7 a | 23.9 ± 0.3 a | 22.7 ± 0.3 a | 26.7 ± 0.1 a | 56.1 ± 1.5 a |
| *Calcium* | *DW* | 12.6 ± 0.1 c | 11.9 ± 0.1 c | 11.9 ± 0.1 c | 12.3 ± 0.2 c | 12.7 ± 0.1 c | 12.0 ± 0.1 c | 16.4 ± 0.2 b | 13.2 ± 0.2 c | 4.53 ± 0.07 c |
|  | *TA* | 22.4 ± 0.3 a | 21.4 ± 0.3 a | 21.0 ± 0.1 a | 21.8 ± 0.2 a | 24.3 ± 0.2 a | 22.2 ± 0.3 a | 23.5 ± 0.2 a | 22.5 ± 0.2 a | 10.1 ± 0.1 b |
|  | *EDTA* | 15.8 ± 0.1 b | 14.9 ± 0.1 b | 15.0 ± 0.1 b | 15.7 ± 0.2 b | 16.0 ± 0.2 b | 17.0 ± 0.1 b | 13.2 ± 0.02 c | 13.8 ± 0.2 b | 12.5 ± 0.1 a |
| *Magnesium* | *DW* | 0.84 ± 0.01 b | 0.82 ± 0.00 c | 0.82 ± 0.01 b | 0.85 ± 0.01 b | 0.90 ± 0.01 c | 0.87 ± 0.01 c | 1.26 ± 0.01 b | 1.02 ± 0.01 c | 0.57 ± 0.01 c |
|  | *TA* | 1.33 ± 0.02 a | 1.32 ± 0.01 a | 1.32 ± 0.01 a | 1.37 ± 0.02 a | 1.43 ± 0.02 a | 1.48 ± 0.02 a | 1.60 ± 0.16 a | 1.85 ± 0.02 a | 0.95 ± 0.01 b |
|  | *EDTA* | 1.32 ± 0.02 a | 1.29 ± 0.01 b | 1.30 ± 0.01 a | 1.34 ± 0.01 a | 1.38 ± 0.02 b | 1.43 ± 0.01 b | 1.39 ± 0.00 ab | 1.35 ± 0.03 b | 1.56 ± 0.03 a |
| *Potassium* | *DW* | 2.14 ± 0.04 c | 2.22 ± 0.09 b | 2.20 ± 0.11 b | 1.94 ± 0.12 b | 1.86 ± 0.18 b | 1.46 ± 0.03 b | 0.71 ± 0.03 a | 0.39 ± 0.00 c | 0.16 ± 0.00 c |
|  | *TA* | 2.41 ± 0.04 b | 2.26 ± 0.06 b | 2.39 ± 0.12 ab | 2.21 ± 0.12 ab | 2.16 ± 0.05a | 1.66 ± 0.03 a | 0.68 ± 0.02 a | 0.50 ± 0.01 a | 0.36 ± 0.00 a |
|  | *EDTA* | 2.56 ± 0.07 a | 2.64 ± 0.05 a | 2.62 ± 0.13 a | 2.36 ± 0.19 a | 2.22 ± 0.03 a | 1.72 ± 0.03 a | 0.32 ± 0.02 b | 0.42 ± 0.00 b | 0.23 ± 0.00 b |
| *Sodium* | *DW* | 1.56 ± 0.02 b | 1.67 ± 0.02 b | 1.65 ± 0.01 b | 1.56 ± 0.03 b | 1.55 ± 0.05 b | 1.47 ± 0.01 b | 1.67 ± 0.01 b | 0.50 ± 0.01 c | 0.18 ± 0.00 c |
|  | *TA* | 1.42 ± 0.02 b | 1.48 ± 0.01 c | 1.48 ± 0.01 b | 1.41 ± 0.04 b | 1.45 ± 0.03 b | 1.40 ± 0.02 b | 1.01 ± 0.00 c | 0.70 ± 0.01 b | 0.65 ± 0.01 b |
|  | *EDTA* | 8.76 ± 0.11 a | 8.71 ± 0.09 a | 8.71 ± 0.22 a | 8.61 ± 0.23 a | 8.72 ± 0.08 a | 8.66 ± 0.25 a | 6.52 ± 0.04 a | 5.12 ± 0.06 a | 4.58 ± 0.02 a |
| *Lactate* | *DW* | 0.95 | 0.66 | 1.20 | 0.79 | 0.52 | 1.15 | 1.52 | 0.75 | 0.65 |
|  | *TA* | 0.60 | 0.80 | 1.29 | 0.79 | 1.25 | 0.93 | 0.82 | 2.43 | 1.69 |
|  | *EDTA* | 0.32 | 1.30 | 0.66 | 0.25 | 1.00 | 1.07 | 1.11 | 1.45 | 1.43 |
| *Acetate* | *DW* | 0.16 | 0.29 | 0.30 | 0.14 | 0.25 | 0.22 | 0.50 | 0.13 | 0.39 |
|  | *TA* | 0.17 | 0.14 | 0.45 | 0.30 | 0.22 | 0.25 | 0.22 | 0.21 | 0.31 |
|  | *EDTA* | 0.11 | 0.24 | 0.34 | 0.24 | 0.12 | 0.22 | 0.25 | 0.55 | 0.40 |
| *Formate* | *DW* | 0.07 | 0.11 | 0.19 | 0.04 | 0.31 | 0.08 | 0.30 | 0.06 | 0.07 |
|  | *TA* | 0.08 | 0.07 | 0.40 | 0.04 | 0.08 | 0.11 | 0.12 | 0.37 | 0.15 |
|  | *EDTA* | 0.05 | 0.11 | 0.12 | 0.08 | 0.07 | 0.08 | 0.12 | 0.15 | 0.11 |
| *Malate* | *DW* | 0.73 | 0.54 | 0.18 | 0.21 | 0.45 | 0.34 | 0.26 | 0.25 | 0.20 |
|  | *TA* | 0.67 | 0.20 | 0.19 | 0.20 | 0.29 | 0.38 | 0.24 | 0.35 | 0.22 |
|  | *EDTA* | 0.62 | 0.22 | 0.19 | 0.19 | 0.24 | 0.53 | 0.09 | 0.23 | 0.00 |
| *Oxalate* | *DW* | 0.14 | 0.23 | 0.17 | 0.11 | 0.12 | 0.16 | 0.38 | 2.65 | 0.44 |
|  | *TA* | 0.22 | 0.18 | 0.19 | 0.24 | 0.13 | 0.14 | 0.33 | 1.54 | 0.46 |
|  | *EDTA* | 0.16 | 0.18 | 0.17 | 0.11 | 0.13 | 0.17 | 0.31 | 0.98 | 0.48 |
| *Fluoride* | *DW* | 0.21 | 0.16 | 0.23 | 0.35 | 0.18 | 0.22 | 0.27 | 0.19 | 0.30 |
|  | *TA* | 0.12 | 0.16 | 0.25 | 0.15 | 0.24 | 0.18 | 0.18 | 0.47 | 0.32 |
|  | *EDTA* | 0.15 | 0.25 | 0.16 | 0.16 | 0.20 | 0.21 | 0.19 | 0.28 | 0.31 |
| *Chloride* | *DW* | 16.88 | 16.87 | 14.40 | 17.13 | 17.39 | 14.23 | 3.83 | 0.20 | 0.47 |
|  | *TA* | 31.17 | 31.34 | 31.70 | 31.36 | 30.85 | 24.85 | 20.63 | 18.12 | 0.93 |
|  | *EDTA* | 22.50 | 21.59 | 21.51 | 20.29 | 19.32 | 17.10 | 7.64 | 0.87 | 0.61 |
| *Sulfate* | *DW* | 2.35 | 2.38 | 1.86 | 2.52 | 5.01 | 1.80 | 3.64 | 2.76 | 4.11 |
|  | *TA* | 1.81 | 1.94 | 1.94 | 1.80 | 1.77 | 1.43 | 2.22 | 6.06 | 2.19 |
|  | *EDTA* | 5.40 | 6.67 | 5.61 | 5.23 | 5.40 | 5.05 | 6.08 | 5.30 | 5.91 |
| *Phosphate* | *DW* | 0.20 | / | / | / | / | / | 0.14 | 0.13 | / |
|  | *TA* | 0.15 | 0.09 | / | / | 0.23 | / | / | 0.16 | / |
|  | *EDTA* | 0.06 | / | 0.31 | / | / | / | / | 0.34 | / |
| *Nitrate* | *DW* | 0.785 | 0.713 | 0.676 | 0.658 | 0.672 | 0.476 | / | / | / |
|  | *TA* | 0.004 | 0.020 | 0.006 | / | 0.020 | 0.003 | / | / | / |
|  | *EDTA* | 1.249 | 1.162 | 1.162 | 1.157 | 1.104 | 0.761 | / | / | / |
| *Ammonium* | *DW* | 0.207 | 0.053 | 0.104 | 0.047 | 0.079 | 0.083 | 0.055 | 0.050 | 0.004 |
|  | *TA* | 0.018 | 0.008 | 0.019 | 0.031 | 0.010 | 0.068 | 0.062 | 0.055 | 0.011 |
|  | *EDTA* | 0.304 | 0.136 | 0.145 | 0.142 | 0.101 | 0.076 | 0.046 | 0.052 | 0.003 |
